# Metaphorical or Straightforward? Comparing the Effectiveness of Different Types of Social Media Advertising

**DOI:** 10.3389/fnins.2022.851729

**Published:** 2022-05-11

**Authors:** Xin Ding, Ping Feng, Jingqiang Wang, Meizhen Lin

**Affiliations:** College of Tourism, Huaqiao University, Quanzhou, China

**Keywords:** metaphorical advertisements, straightforward advertisements, post popularity, visual attention, eye-tracking technology, elaboration likelihood model

## Abstract

The existing studies have analyzed the advertising effects of metaphorical advertisements and straightforward adverts in traditional advertising media. However, their advertising effects on social media are still unclear. To address this issue, this study uses eye-tracking and questionnaires to examine two types of social media tourism advertising—metaphorical and straightforward in posts with both high and low popularity. This within-subject (*n* = 55) experiment was designed to examine the effects of social media tourism advertising types on visual attention and tourism intention and to identify the moderating role of post popularity based on the elaboration likelihood model (ELM). We found that advertising types had no significant effect on visual attention, but metaphorical advertisements increased tourism intention compared with straightforward adverts. Furthermore, we found that the level of post popularity moderated the effect of advertising types on visual attention. Specifically, metaphorical adverts in highly popular posts attracted more visual attention in the advertising text area and in the whole advert. Straightforward adverts in posts with low popularity attracted more visual attention in the advertising text area. This research advances the current literature by exploring the effects of social media tourism advertising types and has implications for managers deciding on strategies for social media tourism marketing.

## Introduction

Metaphorical advertisements are adverts that appeal implicitly through abstract and artistic words (Ang and Lim, 2013). Metaphorical advertisements are widely used in marketing because they can increase communication between consumers and advertisers (Maclnnis et al., 1991), enhance the interest in the advertising (Ang, 2002), and improve the advertising attitude of consumers (McQuarrie and Mick, 1999). Straightforward advertisements, in contrast, appeal explicitly through literal and narrative words (Lagerwerf and Meijers, 2008). Straightforward advertisements have long been used for the launch of new products because they highlight their features and make the information easier to receive (Ziamou and Ratneshwar, 2003). Previous research on tourism advertising in traditional media, such as television, press, and radio, has found that metaphorical advertisements tend to attract more visual attention and promote buying due to the longer processing time and the more positive attitudes of the audience (Phillips and McQuarrie, 2009). Does it mean that metaphorical advertisements are also better on social media? The answer is still uncertain. Unlike traditional tourism advertisements, social media tourism advertisements have the characteristics of “explosion and fragmentation” (Bartschat et al., 2021), and a large amount of advertising information often appears at the same time in the moment of searching. Due to the limited cognitive resources of consumers (Lavie, 1995), it seems that the easier-to-understand straightforward advertisement leaves a deeper impression on consumers than the metaphorical advertisement. Therefore, our research addresses how metaphorical advertisements and straightforward advertisements compare in the context of social media and also explores the boundary conditions influencing this effectiveness.

Based on the elaboration likelihood model (ELM), there are two methods of persuasion, namely, central and peripheral routes (Petty and Cacioppo, 1986). Central route persuasion is that which likely “resulted from a person’s careful and thoughtful consideration of the true merits of the information presented in support of an advocacy.” Peripheral route persuasion is that which more likely “occurs as a result of some simple cues in the persuasion context that induces change without necessitating scrutiny of the true merits of the information presented” (Petty and Cacioppo, 1986). These simple peripheral cues refer to stimuli that can affect attitudes without necessitating processing of the message arguments, such as source expertise, source credibility, and so on (Petty and Cacioppo, 1986). Post popularity is also an important factor in peripheral cues (Chang et al., 2015). Post popularity is defined by the number of online likes, shares, and comments. It is regarded as an important influence on consumers when they are processing posts on social media (Park et al., 2007). When the post popularity is low, consumers’ attitudes and behaviors are mainly influenced by the content of the post, but when the post popularity is high, they are more likely to be influenced by simple cues in the post. In other words, the attitude and behavior of consumers differ depending on the popularity of the post. Therefore, our study takes post popularity as the moderating variable to explore the boundary conditions of advertising types.

Advertising information overload is a feature of social media. The number of advertisements that people are exposed to every day has risen from 2,000 to 5,000 (Ahn et al., 2018). The intangibility of the tourism experience makes the effect of tourism advertising largely dependent on visual attention. Based on this, visual attention has become a key factor for measuring the effectiveness of tourism advertising. A recent study, however, has also suggested that, since actual travel behavior is difficult to measure, tourism intention often becomes the final factor for measuring advertising effectiveness (Weng et al., 2021). Therefore, this study explores the effectiveness of social media tourism advertising types using both visual attention and tourism intention. With the help of eye-tracking technology and questionnaires, we explored the influence of metaphorical vs. straightforward social media advertising on visual attention and tourism intention and the moderating role of post popularity.

## Literature Review and Hypothesis Development

### Tourism Advertising and Visual Attention

Researchers have divided advertisements into metaphorical and straightforward (Lakoff and Johnson, 1980), which are then further divided into textual and pictorial based on their advertising elements (McQuarrie and Phillips, 2005). In earlier studies, the focus has mainly been on pictorial advertisements (Oi̧ehnoviča et al., 2016), but text in tourism adverts is still the focus of research on advertising effectiveness (Adu-Ampong, 2016).

Attention is regarded as a mechanism for additional processing of selective information (Greenwald and Leavitt, 1984), while visual attention “can be thought of as relying on a collection of paintbrushes (neurons) that are trying to paint stimuli (objects in the environment) on a canvas (the visual cortex) so that perceptual processes can interpret the canvas” (Janiszewski et al., 2013). According to the limited attention model, individuals will select their visual attention, that is, they may prioritize the processing of stimulating information according to their own preferences (Lynch and Srull, 1982). There are two selective methods. The first is top-down salient filtering selection, and the second is bottom-up control selection (Pieters and Wedel, 2004). The former belongs to the realm of unconscious automatic selection, which is mainly based on the physical characteristics of the visual stimulus itself (e.g., color, size, brightness, and so on) (Janiszewski, 1998), while the latter belongs to the conscious proactive selection, which is mainly based on the long-term formation of preferences, expectations, and motivations (Itti and Koch, 2001).

In the field of tourism advertising, existing research has mainly explored the influence of advertising elements such as texts, pictures, brand logos, colors, and sizes on visual attention. Results have included the finding that adverts that contain landscape pictures with naturally embedded words (Li et al., 2016), brands logos of the destination (Lourenção et al., 2020), and colors similar to those of the web pages (Chiu et al., 2017) can attract more visual attention. Some studies have also examined the influence of banner advertising types on visual attention and found that static advertisements can first attract the audience’s visual attention, while dynamic advertisements can attract more visual attention from the audience (Hernández-Méndez and Muñoz-Leiva, 2015). Although these studies have been valuable, there are still many research questions that need to be addressed. First, most of these studies have been conducted in the traditional advertising context, investigating the visual attention of billboard, print, and website advertisements, but ignoring the emerging context of social media tourism advertisements. Second, most of the existing research has mainly focused on the influence of advertising elements on visual attention rather than the type of advertising. Therefore, to advance the research, this study explores the effect of social media tourism advertising types (metaphor and straightforward) on visual attention.

The ELM, which provides a general framework for organizing, categorizing, and understanding the basic processes underlying the effectiveness of persuasive communications, is widely used in metaphorical advertising research. Central route and peripheral route persuasion can be seen as opposite extremes of a continuum (Hardy et al., 2018). Central route persuasion requires greater effort and leads to the largest behavioral effect, while the peripheral route does not involve much cognitive effort (Petty and Cacioppo, 1986). Metaphorical advertisements are abstract and creative. They can activate more associations in a consumer’s semantic memory, making them call on transcendental knowledge and logical thinking to carefully scrutinize advertisements, which requires more cognitive efforts (Lee et al., 2019). In contrast, straightforward advertisements are easy to understand and take less cognitive effort. When a message is presented to individuals in different contexts and situations, the way recipients process the message will vary according to how much cognitive effort they devote to that message (Hardy et al., 2018). Therefore, it can be speculated that consumers will adopt the central route when processing metaphorical advertisements and the peripheral route when processing straightforward advertisements. Visual attention is the key to information processing (Just and Carpenter, 1980), so the allocation of cognitive resources can be taken as the allocation of visual attention. Since metaphorical advertisements require more processing, they will require more visual attention. Therefore, we proposed the following hypothesis:

H1: Compared with straightforward advertisements, metaphorical advertisements increase visual attention.

### Tourism Advertising and Tourism Intention

Tourism intention is a popular indicator for assessing the effectiveness of tourism advertisements (Weng et al., 2021). The stronger the tourism intention, the more likely the tourists engage in actual tourism behavior. Tourism advertisements stimulate tourism intention by presenting destination-related information in pictures, words, videos, and so on (Walters et al., 2007). Existing research mainly explores the influence of advertising elements, such as text or pictures, and advertising media (print, video, virtual reality, and so on) on tourism intention. Findings have shown that, compared with tourism advertisements without text, tourism advertisements composed of both text and pictures stimulate higher tourism intention (Tercia et al., 2020). Print advertisements stimulate higher tourism intention than VR advertisements, and video advertisements stimulate higher tourism intention than print advertisements (Guerrero-Rodríguez et al., 2020; Weng et al., 2021). Some studies have also explored the influence of subjective and objective language styles on consumer behavior in the context of hotel advertising. The results showed that subjective advertisements, characterized by more personalized features, resulted in higher click-through rates, while objective advertisements with greater brand consistency led to a higher advertising conversion rate (Huang and Liu, 2021).

Even though extensive studies have focused on the influence of tourism advertisements on tourism intention, few studies have studied social media tourism advertisements. The relative effectiveness of straightforward and metaphoric advertising is also highly relevant to a field where abstract and creative words might be assumed to better portray a destination as a good utopia (Phillips et al., 2021). According to Tao et al. (2022), straightforward advertisements are generally considered to be more conducive to encouraging consumers to make decisions since they can directly display the functions of products. The question of which style of advertisement stimulates higher tourism intention still needs further exploration.

Compared with the peripheral route that relies on simple cues for information processing, the central route that relies on thoughtful consideration can produce a more enduring persuasion (Petty and Cacioppo, 1986). Furthermore, the ELM suggests that “the final consequence of the route to persuasion is that attitudes formed *via* the central route should be more resistant to counterpropaganda than attitudes formed *via* the peripheral route.” In other words, individuals who process information through the central route are more receptive to marketing messages than individuals who process information through the peripheral route (Petty and Cacioppo, 1986; Chang et al., 2020). Yang (2015) found that the higher the level of elaboration, the higher the purchase intention. Chang et al. (2020) found that, compared with the peripheral route (post aesthetics and post popularity), consumers in the central route (information completeness and information accuracy) had a more positive attitude toward advertisements and higher purchase intention. From this, it is suggested that individuals adopting the central route will have higher behavioral intentions. Therefore, we proposed the following hypothesis:

H2: Compared with straightforward advertisements, metaphorical advertisements increase tourism intention.

### The Moderating Role of Post Popularity

Post popularity refers to the number of online likes, shares, and comments (Chang et al., 2020). It is a critical factor for increasing the persuasiveness of a post. The more popular the post, the more worthy of attention and the higher the credibility of the post. Jin and Muqaddam (2018) found that the level of post popularity could moderate the influence of post types on perceived narcissism, and Mekawie and Hany (2019) found that the level of post popularity could moderate the influence of product types on purchase intention. Our study speculated that post popularity would moderate the influence of advertising types on visual attention and tourism intention. For this study, we set the high post popularity to 838,796 likes, 592,398 shares, and 150,171 comments, while the low post popularity to 228 likes, 4 shares, and 12 comments.

Post popularity moderates the effect of advertising types on visual attention. High post popularity means that a large number of users pay attention to the post, and based on the herding effect (Banerjee, 1992), other users will also pay attention to the post (Mattke et al., 2020). Metaphorical advertisements can stimulate more elaboration in processing, so will get more visual attention than straightforward advertisements in posts with high popularity. Posts with low popularity attract fewer viewers and less attention, so the herding effect means that other users will also reduce their attention to the post (Mattke et al., 2020). Since processing capacity is limited, individuals may prefer to allocate it to simple cognitive tasks (Cabañero Gómez et al., 2021). The implication is that viewers are more willing to process straightforward advertisements than metaphorical advertisements in low popularity posts. In other words, straightforward advertisements gain more visual attention from audiences. Therefore, we proposed the following hypothesis:

H3: Post popularity moderates the effects of advertising types on visual attention. Metaphorical adverts attract more visual attention in highly popular posts and less attention in posts with low popularity than straightforward adverts.

Post popularity can also moderate the effect of advertising types on tourism intention. Highly popular posts lead to discussion, most of which is around sharing experience and knowledge. These discussions are spontaneous, authentic, and nonprofit, which can increase an audience’s interest in the products and improve the advertising effect (Daugherty et al., 2008). In highly popular posts, metaphorical advertisements that need careful and thoughtful scrutiny are often more persuasive than straightforward adverts (Chang and Yen, 2013). Low post popularity means less discussion, which increases uncertainty and perceived risk, leading to a negative effect on purchasing. Straightforward advertisements that focus on product functions and features can, however, lessen the perceived risks and promote purchase. Therefore, we proposed the following hypothesis:

H4: Post popularity moderates the effect of advertising types on tourism intention. Metaphorical advertisements stimulate higher tourism intention in highly popular posts and lower tourism intention with low popularity than straightforward adverts.

## Research Methodology

### Experimental Design

The hypotheses proposed in this study were tested using a 2 × 2 within-subject design, with two advertising types (metaphorical vs. straightforward) and two levels of post popularity (high vs. low). The experimental stimuli were posts on Sina Weibo featuring tourism advertisements. Four destinations were selected for the experiment, and each destination was set up with four conditions (high/low popularity metaphorical advertisements and high/low popularity straightforward advertisements). In all, there were 16 posts. To eliminate the sequential effect, a multifactor Latin square design was adopted. The 16 posts were divided into four groups, each with four adverts, and each advert corresponding to an experimental condition. Each participant was randomly assigned to one of the groups, and after browsing a post, they answered questions about their tourism intention. The scale for tourism intention consisted of six items adapted from Zeithaml et al. (1996) using a Likert 7-point scale.

• “I’m looking forward to traveling to that destination.”
• “I’d like to travel to that destination.”
• “For this trip, I will choose that destination first.”
• “I have been looking forward to traveling to that destination.”
• “I will recommend my relatives and friends to travel to this destination.”
• “I will travel to the destination with my family and friends.”

The mean of tourism intention was 4.675, the SD was 1.147, and the Cronbach’s α was 0.924. When the participants finished the eye movement experiment, they were asked to evaluate the stimulus. This was to complete the manipulation test of the experimental materials.

### Experimental Stimuli

#### Sina Weibo

We chose Sina Weibo for the following reasons. First, Sina Weibo is a huge social media platform (similar to Facebook and Instagram) that integrates life and entertainment in China. At the end of September 2021, the platform had 573 million monthly active users, 80% of them born after 1995 (Weibo, 2021a). The platform’s demographics also map well to those of tourism, given the tourists are getting younger (Torres-Moraga et al., 2021). Second, according to the Weibo’s User Development Report in 2020, tourism topics topped the list of life topics on Sina Weibo, and there were several tagged themes with a discussion volume of over 100 million (e.g., “Travelling with Ding Zhen,” “Snow in Beijing Summer Palace”) (Weibo, 2021b). Sina Weibo has become an important platform for tourists to share and discuss tourism experiences.

#### Pretest 1: Choice of Destinations

How well-known a destination is affects visual attention and tourism intention. We chose moderately well-known destinations for the study that showed no significant difference between them in terms of familiarity. To ensure the consistency and equivalence of the content of the adverts, both cultural and natural landscape destinations were selected from the Ministry of Culture and Tourism of China’s list of National AAAAA Tourist Attractions. We excluded the top 20 destinations from the list of destinations released by Ctrip (the largest travel website in China). We ultimately chose 16 destinations: Jin Zhong, Bao Ding, Chang Chun, Xin Zhou, Ordos, Ning Bo, Yan Tai, Wu Xi, Huang Shan, Yi Chang, Wei Hai, Zhang Jiajie, Shang Rao, Yue Yang, Fo Shan, and Aba Tibetan Autonomous Prefecture. We designed a questionnaire to measure the familiarity with and attractiveness of the 16 destinations. One item was used to measure the attractiveness of each destination—“I think this travel destination is attractive,” and four items from the Baloglu (2001) scale were used to measure the familiarity with each destination:

• “I have heard this destination introduced by my relatives or friends.”
• “I have seen or heard about this tourism destination in relevant media.”
• “I specifically searched for the tourism destination through relevant media.”
• “How many times have I visited the destination?”

The results showed that, among the 16 destinations, four destinations—Jin Zhong, Bao Ding, Yan Tai and Chang Chun—ranked in the middle position, and there was no significant difference in familiarity with them (*M*_*Jin Zhong*_ = 2.93, *M*_*Bao Ding*_ = 3.27, *M*_*Yan Tai*_ = 3.29, *M*_*Chang Chun*_ = 3.35; *p*’s > 0.05) and attractiveness (*M*_*Jin Zhong*_ = 3.29, *M*_*Yan Tai*_ = 3.40, *M*_*Chang Chun*_ = 3.69, *M*_*Bao Ding*_ = 3.77; *p*’s > 0.05). Based on this, we chose these four cities as the destinations for the study.

#### Pretest 2: Advertising Types and Post Popularity

We designed two types of advertising (metaphorical and straightforward) and two levels of post popularity (high/low) for each destination. We collected nine landscape photos for each destination, which is the maximum allowed for a post. We used Photoshop to give all the images the same tone and set all sizes to 1,920 × 1,080 pixels. We designed metaphorical and straightforward advertising text for each destination, giving us eight adverts. These were then placed with high and low popularity posts, giving 16 advertisements in total. Based on our definitions for high and low popularity, the posts used had 838,796 likes, 592,398 shares, and 150,171 comments, and 228 likes, four shares, and 12 comments, respectively. The 16 designed adverts were published through Sina Weibo, and 46 participants were invited to evaluate them to determine whether they were metaphorical or straightforward. The question from Delbaere et al. (2011) was used to define this, using a Thurstone scale—“Do you think the ad is explicit and factual, or abstract and artistic?” (1 = explicit and factual, 7 = abstract and artistic). The results showed that there were significant differences between metaphorical advertisements and straightforward advertisements in Jin Zhong (*M*_*straight forward*_ = 2.85, *M*_*metaphorical*_ = 4.89, *p* < 0.001), Bao Ding (*M*_*straight forward*_ = 3.02, *M*_*metaphorical*_ = 4.96, *p* < 0.001), Yan Tai (*M*_*straight forward*_ = 2.96, *M*_*metaphorical*_ = 5.33, *p* < 0.001), and Chang Chun (*M*_*straight forward*_ = 3.46, *M*_*metaphorical*_ = 5.25, *p* < 0.001) (refer to Table A1 of Appendix A).

### Participants

We selected college students as participants in the experiment. Using a single sample group is beneficial to avoid the interference of population characteristics in the experiment (Wang and Hung, 2019). More importantly, the demographic characteristics of college students are similar, and the internal differences are small, which is conducive to data analysis and comparison. The sample was also more representative and ecologically valid because 16- to 25-year-olds account for 80% of active users on Sina Weibo (Weibo, 2021a).

We recruited 60 college students with experience on Sina Weibo to participate in the experiment. After eliminating five invalid data, 55 valid samples were retained (28 women and 27 men with an average age of 22.37). We used G-power 3.1 for sample size estimation. The results showed that the sample size required for this study was *n* = 34. Therefore, using 55 samples was statistically in line with the experimental requirements of this study.

### Procedure

**FIGURE 1:**
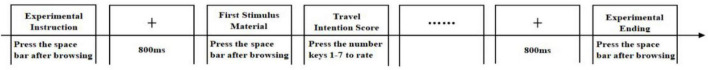
Flowchart of eye movement experiment for a single trial.

A calibration test was conducted before the experiment to ensure the average deviations in two directions were < 1 (Li et al., 2020). The eye-tracker (Eyeso EC80) at a sampling rate of 30FPS was attached beneath the laptop screen (13.3-inch monitor) with a resolution of 1,920 × 1,080 pixels. Participants were positioned approximately 70 cm from the monitor. The default calibration settings of the eye-tracker were used to run the calibration. Since instruction can affect viewing patterns (Müller et al., 2012), we told the participants what they needed to imagine through the experiment instruction. We conducted a series of practical experiments to help the participants get familiar with the operation and process of the experiment and avoid operational errors. The formal experiment included four stages, namely, reading the experiment instructions, seeing a plus sign in the center of the screen, browsing social media tourism advertisements, and scoring the tourism intention items. The flowchart of the eye movement experiment for a single trial is shown in Figure 1.

## Data Analysis

### Eye Movement Indicators

Area of interest (AOI) is a basic unit of visual attention (Scott et al., 2019). Its scope is defined by the research purpose (Bialkova and van Trijp, 2011). Our study sets both the text area and the whole advert as the interest areas. A fixation is defined as “the brief amount of time when the eyes stay temporarily still and gaze at a specific point of the visual field” (Rayner, 2009). The number of fixations (FC) and fixation duration (FD) are the most common indicators in eye movement experiments (Bialkova and van Trijp, 2011). The FC is a reliable indicator of visual attention, which reflects how many times the audiences’ eyes paused on an area (Li et al., 2016). The FD reflects the time that an audience spends on one fixation (Li et al., 2016). In line with previous study, such as Hernández-Méndez and Muñoz-Leiva (2015), Li et al. (2016; 2020), we selected the FC and the FD to test the participants’ visual attention.

### Manipulation Checks

After the experiment, the subjects were asked to score their perception of the advertising types (“Do you think the advertisements are explicit and factual, or abstract and artistic?” 1 = explicit and factual, 7 = abstract and artistic) and post popularity (“Do you think the ads is low popularity, or high popularity?” 1 = low popularity, 7 = high popularity) from 1 to 7. The results show that there were significant difference between straightforward advertisements and metaphorical advertisements of the four destinations: Jin Zhong (*M*_*straight forward*_ = 2.49, *M*_*metaphorical*_ = 4.76, *t* (108) = -8.81, *p* < 0.001), Bao Ding (*M*_*straight forward*_ = 2.76, *M*_*metaphorical*_ = 4.58, *t* (108) = 6.98, *p* < 0.001), Yan Tai (*M*_*straight forward*_ = 3.01, *M*_*metaphorical*_ = 3.94, *t* (108) = 6.70, *p* < 0.001), and Chang Chun (*M*_*straight forward*_ = 2.72, *M*_*metaphorical*_ = 4.49, *t* (108) = 6.72, *p* < 0.001). Additionally, there was also a significant difference between high post popularity and low post popularity (*M*_*high*_ = 5.20, *M*_*low*_ = 3.39, *t* (108) = 5.51, *p* = 0.00). These results indicated that intended stimuli were manipulated successfully in the experiments.

### Descriptive Statistical Analysis Results

The experiment measured the FC and FD of the participants for the whole posts and the text area. The mean and standard deviation of the FC in the whole adverts were 55.050 and 28.141, and the FD was 25,073.372 ms and 14,431.395 ms. The mean and standard deviation of the FC in the text area were 37.027 and 21.275, the FD was 14,417.668 ms and 10,022.356 ms. Table 1 shows the descriptive statistics of the eye movement indicators in the whole tourism advertisements and the text area under the high and the low post popularity.

**TABLE 1:** Descriptive statistics of eye movement data.

		High-popularity post				Low-popularity post			
		The whole advert		The text area		The whole advert		The text area	
		FC	FD	FC	FD	FC	FD	FC	FD
MAs	M	60.927	27,378.618	39.982	15,907.236	53.655	23,864.236	34.618	12,984.945
	SD	33.084	17,037.004	24.446	11,534.261	24.221	12,812.575	19.261	8,422.605
SAs	M	50.655	22,849.236	35.582	14,069.109	54.964	26,201.400	37.927	14,709.382
	SD	27.034	13,965.714	19.758	10,374.576	27.255	13,488.223	21.422	9,757.983

*MA, metaphorical advertisements; SA, straightforward advertisements. Photos displayed were obtained from Baidu (https://image.baidu.com/).*

### Hypothesis Testing

For social media tourism advertising types referring to Sina Weibo, a 2 (tourism advertising type: metaphor vs. straightforward) × 2 (post popularity: high vs. low) repeated ANOVAs were performed regarding the visual attention and tourism intention. The results are shown in Table 2.

**TABLE 2:** Results of repeated measures of two-factor ANOVA.

			Visual attention							
			The whole advert				The text area of the advert			
	Tourism intention		FC		FD		FC		FD	
	MS	F	MS	F	MS	F	MS	F	MS	F
AT	16.382	12.686**	1,104.768	3.457	66.080	0.726	16.364	0.184	17.773	0.010
PP(H/L)	2.062	2.771	120.768	0.265	0.362	0.003	125.255	0.762	71.605	1.627
AT × PP	0.180	0.183	1,844.405	6.829*	648.305	6.984*	817.164	5.227*	174.513	7.364**

*AT, advertising types; PP, post popularity.*

The fixation duration (FD) was measured in seconds, *p < 0.05, **p < 0.01.

Results of repeated ANOVAs indicated that there was no significant difference in the FC and the FD for the whole adverts and the text area between metaphorical advertisements and straightforward advertisements (*p*s > 0.05) for visual attention. Thus, hypothesis 1 is not supported.

Results of repeated ANOVAs indicated that tourism intentions were significantly higher when processing the metaphorical advertisements than straightforward advertisements (*M*_*metaphorical*_ = 4.959, *M*_*straightforward*_ = 4.413, *F*(1,54) = 12.686, *p* = 0.001 < 0.01). Thus, hypothesis 2 is supported.

**FIGURE 2:**
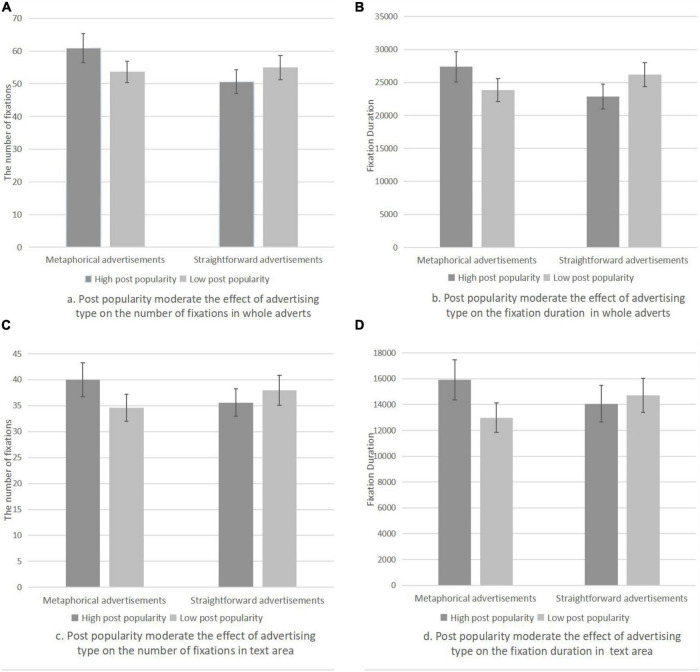
Peripheral cues moderate the effect of advertising types on visual attention.

Results of repeated ANOVAs showed that there was no significant interaction of post popularity and advertising type on tourism intentions (*F*(1, 54) = 0.183, *p* = 0.67). Thus, hypothesis 3 is not supported. However, post popularity moderated the relationship between advertising types and the FC (*F*(1, 54) = 6.829, *p* = 0.012 < 0.05) and FD (*F*(1, 54) = 6.984, *p* = 0.011 < 0.05) for the whole adverts (Figures 2A,B). Post popularity also moderated the relationships between advertising types and the FC in the text area (*F*(1, 54) = 5.227, *p* = 0.026 < 0.05) and the FD (*F*(1,54) = 7.364, *p* = 0.009 < 0.01) (Figures 2C,D). Thus, hypothesis4 is preliminarily verified.

To analyze the influence of advertising types on visual attention and tourism intention at every level of post popularity, simple effect analysis was further employed. As presented in Table 3, results showed that with high post popularity, the influence of advertising types on the FC and FD was significant for the whole adverts and marginally significant for the textual area (*F*_*FCP*_(1, 54) = 10.104, *p*_*FCP*_ = 0.002 < 0.01; *F*_*FDP*_(1, 54) = 8.831, *p*_*FDP*_ = 0.004 < 0.01; *F*_*FCT*_(1, 54) = 3.894, *p*_*FCT*_ = 0.054; *F*_*FDT*_ (1, 54) = 3.873, *p*_*FDT*_ = 0.054). With low post popularity, there was no significant difference for the FC and FD between metaphorical advertisements and straightforward advertisements in either the whole adverts or the text area. However, the effect of straightforward advertisements on the FD was significant in text areas [*F*(1, 54) = 4.603, *p* = 0.036 < 0.05].

**TABLE 3:** Pairwise comparisons.

		High-popularity posts		Low-popularity posts	
		Metaphorical adverts	Straightforward adverts	Metaphorical adverts	Straightforward adverts
	Dependent variable	M (SD)	M (SD)	M (SD)	M (SD)
Visual attention	FCP	60.927 (4.461)	50.655 (3.645)	53.655 (3.266)	54.964 (3.675)
	F	10.104**		0.156	
	FDP	27,378.610 (2,297.269)	27,378.618 (2,297.269)	23,864.236 (1,727.639)	22,849.236 (1,883.136)
	F	8.831**		1.254	
	FCT	39.982 (3.296)	35.582 (2.664)	34.618 (2.597)	37.927 (2.889)
	F	3.894		2.769	
	FDT	15,907.236 (1,555.279)	14,069.109 (1,398.908)	12,984.945 (1,135.704)	14,709.382 (1,315.766)
	F	3.873		4.603*	

*p < 0.05, **p < 0.01.

*FCP, the number of fixations in whole adverts; FDP, fixation duration in whole adverts; FCT, the number of fixations in text areas; FDT, fixation duration in text areas.*

In summary, with high-popularity posts, metaphorical advertisements attracted more visual attention for the whole area and the text area than straightforward advertisements. With low popularity posts, straightforward advertisements attracted more visual attention to the text area than metaphorical advertisements. Thus, hypothesis 4 is further supported.

## Conclusion and Discussion

### Conclusion and Implication

By applying eye-tracking experiments and questionnaires, this research aimed to investigate the influence of social media advertising types on visual attention and tourism intention, considering levels of post popularity. We used tourism advertisements with posts on Sina Weibo. The results showed that the type of social media advertising had no significant effect on visual attention. This is inconsistent with previous research into traditional advertising only, which suggested that metaphorical advertisements attracted more visual attention. This may be because, in the context of social media, most users will encounter tourism advertisements when browsing (i.e., information encounters) as well as during purposeful information searches. At the moment of encounter, users tend to scan quickly to process what they see Jeong et al. (2011), so neither metaphorical advertisements nor straightforward can gain more visual attention.

We also found that, based on the theoretical framework of ELM, metaphorical advertisements stimulated higher tourism intention. The reason for this is that, compared with the peripheral route that relies on simple cues for information processing, the central route relies on thoughtful consideration and can produce more enduring persuasion (Petty and Cacioppo, 1986). We also found that the level of post popularity can moderate the influence of social media advertising types on visual attention. For high-popularity posts, metaphorical advertisements attract more visual attention than straightforward advertisements, both for the whole advert and the text area. Posts with low popularity, however, showed no significant difference in the visual attention between the advertising types for the whole advert, but more visual attention for straightforward advertisements in the text area. These findings are explained by the fact that metaphorical advertisements in high-popularity posts stimulate more elaborate processing than straightforward advertisements. In low-popularity posts, the subjects want specific and clear information to reduce perceptual uncertainty. In this case, straightforward advertisements featuring the product functions gain more visual attention.

The main theoretical contributions of this study are as follows. First, previous studies on tourism advertising rarely discuss the effects of different advertising types based on the style of expression and rarely discuss the visual attention through eye movement experiments. Exploring the context of social media advertising, this study examines the visual attention of metaphorical advertisements and straightforward advertisements in the whole advert and the text area, which fills the gap in existing research into tourism advertising. In addition, this study reveals a novel finding that is different from traditional tourism advertising research. Most previous studies suggest that metaphorical advertisements stimulate more visual attention than straightforward advertisements. However, we found that this conclusion was not valid in the social media context. Additionally, we found that the type of advertising only had a significant impact on visual attention when moderated by the level of post popularity. This is an interesting finding which supplements existing tourism advertising research.

Second, the ELM has rarely, if ever, been applied to studies of tourism advertising. Our application of the ELM demonstrates not only how the advertising types influence visual attention and tourism intention in social media marketing but also how post popularity influences these. This verifies the power of ELM to predict and explain the effect of tourism advertisements and expands the scope for applying the ELM. Petty and Cacioppo (1986) defined source expertise and source credibility as peripheral cues when they first proposed the ELM. As a result, scholars have tended to consider only these two factors as peripheral cues. It was not until Yoo et al. (2016) verified interactivity and accessibility as peripheral cues that this view was changed. Our research takes this further by adding post popularity (number of likes, shares, and comments) as peripheral cues.

Third, in previous studies, tourism advertising effectiveness has been mostly assessed through self-reporting, making the accuracy of the measurement controversial (Wang et al., 2020). In addition, although a few scholars have used eye-tracking experiments to conduct tourism advertising research, most of these studies have ignored theoretical support (Lever et al., 2019), or just analyzed the visual pattern of participants but ignored the subjective intention prediction. This study builds a theoretical framework using the ELM and employing eye movement experiments and questionnaires to test hypotheses, extending the line of research on visual processing in the tourism field.

This study also makes some practical suggestions for tourism marketing. Results indicate that, in social media, advertising types have no significant influence on visual attention, except when moderated by post popularity. Therefore, post popularity is the obvious key to influence visual attention. Destinations could improve post popularity by creating hot topics that attract visual attention. Specifically, marketers can combine social media to jointly create hot themes. Hot themes usually appear on the hotlist, which can attract users to click and browse, thus improving post popularity. Encouraging users to interact (i.e., likes, shares, and comments) by lottery (i.e., cash, discount coupons, free air tickets, etc.) is a common way to improve post popularity. Likes and comments can increase the activity of posts, while shares can make more people interact with posts. Inviting key opinion leaders to promote destinations on social media is also a good strategy. Opinion leaders usually have a large number of loyal fans, and destination promotion can attract these fans to interact, thus increasing post popularity.

Results showed that, compared with straightforward advertisements, metaphorical advertisements increased people’s tourism intention. Therefore, destinations should increase the exposure of metaphorical advertisements. Specifically, marketers should harness the resource of users, whose shares are more credible and convincing than the information officially released by the destination (Huerta-Álvarez et al., 2020). Marketers could post metaphorical tourism advertising design activities on social media and invite all users to participate. Every user on social media can freely generate, process, and publish information. Inviting a large number of users to participate in the advertising design activity could increase the spread effect of advertisements and credibility. Marketers should also cooperate with influencers (i.e., online celebrities) to conduct topic marketing. In 2020, there were many tourism-tagged themes with over 100 million discussions on Sina Weibo (Weibo, 2021b). These themes usually start with an influencer’s post. When the post garners more likes, shares, and comments through interaction among fans, other influencers follow and post on the topic too. In this way, the communication effect of advertisements snowballs.

### Limitations and Future Research

There are some limitations that need to be acknowledged. First, this study controls the types of pictures and only explores the effect of advertising types from the perspective of the expressive style of the text. Future research could explore the influence of visual metaphors on advertising effectiveness from the perspective of picture styles. Second, taking our sample from college students limits generalizability, and future research could expand the sample range. Finally, this research focuses on static advertising. With the popularity of video marketing, the effect of video tourism advertising on visual attention and tourism intention is also worthy of future research.

## Data Availability Statement

The data generated for this study are available on request to the corresponding author.

## Ethics Statement

This study was reviewed and approved by the Ethics Committee of the Neurotourism Laboratory at Huaqiao University. All participants provided written informed consent to participate in this study.

## Author Contributions

XD and PF made substantial contributions to the work, participated in all aspects of the manuscript, conducted the experiment, analyzed the data, and wrote the manuscript. JW participated in the data acquisition and data interpretation stage. ML oversaw the study and managed every part of the research.

## Conflict of Interest

The authors declare that the research was conducted in the absence of any commercial or financial relationships that could be construed as a potential conflict of interest.

## Publisher’s Note

All claims expressed in this article are solely those of the authors and do not necessarily represent those of their affiliated organizations, or those of the publisher, the editors and the reviewers. Any product that may be evaluated in this article, or claim that may be made by its manufacturer, is not guaranteed or endorsed by the publisher.

## Appendix

**TABLE A1:** Experimental stimulus materials.
